# Germline duplication of *MYCN* predisposes to childhood embryonal tumours

**DOI:** 10.1016/j.ebiom.2026.106132

**Published:** 2026-01-31

**Authors:** Catherine A. Taylor, Philippa May, Thomas J. Stone, Munaza Ahmed, Tanzina Chowdhury, Deborah A. Tweddle, Shaun Wilson, Ken Hanscombe, J. Ciaran Hutchinson, Jessica C. Pickles, Neil J. Sebire, Thomas S. Jacques

**Affiliations:** a Developmental Biology and Cancer Research and Teaching Department, UCL Great Ormond Street Institute of Child Health, 30 Guilford Street, London, UK; b Department of Histopathology, Great Ormond Street Hospital for Children NHS Foundation Trust, Great Ormond Street, London, UK; c Department of Haematology and Oncology, Great Ormond Street Hospital for Children NHS Foundation Trust, Great Ormond Street, London, UK; d Royal Brompton and Harefield Hospitals, Guy's and St Thomas' NHS Foundation Trust, London, UK; e Specialist Integrated Haematology and Malignancy Diagnostic Service, Great Ormond Street Hospital for Children NHS Foundation Trust, Great Ormond Street, London, UK; f North East Thames Regional Clinical Genetics Service, Great Ormond Street Hospital, Great Ormond Street, London, UK; g Wolfson Childhood Cancer Research Centre, Translational & Clinical Research Institute, Newcastle Centre for Cancer, Newcastle University, Newcastle upon Tyne, UK; h Paediatric Haematology/Oncology Department, John Radcliffe Hospital, Oxford University Hospitals NHS Foundation Trust, Oxford, UK; i Genomics England, London, UK; j Population, Policy and Practice Research and Teaching Department, UCL Great Ormond Street Institute of Child Health, 30 Guilford Street, London, UK

**Keywords:** Whole genome sequencing, Hereditary cancer syndromes, Neuroblastoma, Wilms tumour, MYCN protein

## Abstract

**Background:**

Neuroblastoma and Wilms tumour (WT) are common childhood embryonal malignancies. Germline 2p24 duplication has been reported in several cases of neuroblastoma and WT, either as part of a larger 2p duplication or as a microduplication involving just 2p24.3. Although the larger duplications involve many genes, including *ALK*, the microduplications have been localised to a region including *MYCN* and *DDX1*.

**Methods:**

We analysed Whole Genome Sequence data from adults and children sequenced for various indications. We utilised a workflow to extract structural and copy number variants, filtered to include duplications or gains of 2 kb–20 Mb, including these loci, followed by manual inspection in IGV. Associations were assessed using Fisher's exact test. Penetrance was estimated by Bayesian calculation of the conditional probability of disease.

**Findings:**

Among 113,431 genomes, there were 6 participants with a microduplication that included the *MYCN* locus. Of these, two had a diagnosis of WT and one of neuroblastoma. The 2p24.3 microduplication was therefore identified in 3/197 with a definite history of WT/neuroblastoma and 3/113,234 without such a history (p < 0.0001). Penetrance is estimated to be 13%. Twelve participants were identified with a 2p24.3 microduplication that included the *DDX1* locus but not *MYCN*, none of whom received a diagnosis of a childhood embryonal tumour.

**Interpretation:**

We have shown that 2p24.3 microduplications that include *MYCN* predispose to childhood embryonal tumours and should be routinely assessed when WT or neuroblastoma predisposition is suspected. We have also shown that there does not appear to be any increased incidence of childhood tumours when *DDX1* alone is duplicated.

**Funding:**

UCL Great Ormond Street Institute of Child Health Child Health Research CIO PhD Studentship, 10.13039/501100002203Brain Tumour Charity, 10.13039/501100001273Children with Cancer UK, 10.13039/501100001279Great Ormond Street Hospital Children's Charity, Olivia Hodson Cancer Fund, 10.13039/501100000289Cancer Research UK and the 10.13039/501100000272National Institute for Health Research.

**Research in context**

**Evidence before this study**

Neuroblastoma and Wilm's tumour are common childhood cancers, with familial tumours accounting for 1–2%. The underlying genes responsible can be identified in 85% and 33% of familial cases, respectively, leaving many families without a genetic cause when using existing gene panels. Germline pathogenic variants can also be found in apparently sporadic cases, where there are no other family members with that tumour. This occurs either if the child has a *de novo* mutation, newly occurring in that child, or because the penetrance is such that not everyone with that gene variant will develop childhood tumours.

Neuroblastoma and Wilm's tumour frequently have somatic alterations of *MYCN* as a driver, usually as either an amplification or a gain. There have been previous case reports of neuroblastoma and Wilm's tumour occurring in the context of germline duplications on chromosome 2, in a multi-gene region, which includes *MYCN* and *DDX1,* and also *ALK* in the cases of larger duplications.

A recent guideline paper from the AACR childhood cancer predisposition workshop concluded that there was sufficient evidence for a child to be eligible for neuroblastoma and Wilm's tumour screening if a germline 2p24.3 microduplication was identified. However, it was acknowledged that the gene responsible for the predisposition and its penetrance were unknown at that time.

We searched PubMed using the terms “Neuroblastoma OR Wilm's tumour” AND “MYCN” AND “germline OR constitutional”. All abstracts were reviewed and yielded 4 reports of germline microduplications and some further reports of germline partial trisomies of 2p, found in children diagnosed with either neuroblastoma or Wilm's tumour. All reports of microduplications were included for analysis. The microduplications were all identified on microarray, karyotyping and/or fluorescent in situ hybridisation, and all included both the *MYCN* and *DDX1* genes.

**Added value of this study**

This study set out to test the hypothesis that microduplications of 2p24.3 predispose to childhood embryonal tumours, and further that, specifically, it is the *MYCN* gene duplication that is responsible. To test this, we used the United Kingdom National Genomic Research Library, a large library of whole-genome sequencing data, which provides much higher resolution than the technologies used in the previously reported cases. We show that 2p24.3 microduplications that include *MYCN* predispose to developing neuroblastoma and Wilm's tumour. In our study, 2p24.3 microduplication penetrance is estimated as 13%, which is sufficient to recommend screening by national and international guidelines. We show that it is the *MYCN* gene rather than *DDX1* responsible for the predisposition and that *DDX1* duplication alone does not increase the risk of childhood embryonal tumours. We also demonstrate that the somatic *MYCN* amplification occurring in the case of *MYCN*-amplified neuroblastoma arises at the exact breakpoints of the germline duplication, which has not been shown before.

**Implications of all the available evidence**

We recommend that germline duplication of *MYCN* is considered for inclusion in the germline panels used when testing for germline predisposing variants in childhood neuroblastoma or Wilm's tumour. Children identified would be eligible for screening programmes, which would aim to detect tumours earlier and smaller, and ultimately improve outcomes.

## Introduction

Cancers in childhood often have very different underlying driving processes in comparison to those in adults. The embryonal tumours most commonly occur in early childhood and are frequently driven by aberrant developmental pathways.^1^ As well as a growing understanding of the somatic variants and processes that drive childhood cancers, there has also been increasing awareness of the germline variants that can predispose to childhood tumours. These predisposing germline variants are broadly related to DNA damage repair pathways, cell cycle control/signalling, and developmental pathways.^2^ Identifying patients with an underlying germline predisposition is crucial, as it can enable the identification of family members at risk, refine subsequent follow-up and screening, and impact treatment decisions, particularly the indication for nephron-sparing surgery.

Neuroblastoma and Wilms tumour (WT) are two of the most common childhood embryonal malignancies. Neuroblastoma accounts for 6% of childhood cancer cases but 12% of deaths. Half of neuroblastomas are high-risk, with a 5-year event-free survival ∼50%.^3^ WT is the most common form of childhood kidney cancer. Kidney tumours represent 5% of all childhood cancers, of which 90% are WT.^4^ The overall survival is excellent, with over 90% at 5-years. However, the <10% of patients with bilateral tumours, who are more likely to have an underlying germline predisposition, have a survival rate of <80% and are at significant risk of chronic renal impairment because of treatment.^5^

There are known genetic predispositions for both neuroblastoma and WT. These include variants in *ALK*^6^ and *PHOX2B*^7^ for neuroblastoma (accounting between them for around 85% of familial tumours), and in *WT1, REST, TRIM28* and at 11p15 for WT (combined accounting for around 25% of familial tumours).^8^^,^^9^ Variants in these genes/regions are identified when patients are tested as a result of a variant previously identified within their family, because there is a high clinical suspicion for an underlying germline predisposition, or as part of agnostic Whole Genome Sequencing (WGS). Despite these, and other, known predispositions,^10^ in many familial cases, no variant is identified, and it is well-recognised that further inherited predispositions have yet to be identified for both cancer types.^8^^,^^11^ 3–4% of patients with neuroblastoma and 2% of patients with WT have a family history suggesting a possible predisposition, although a specific genetic cause cannot be found in up to 15% of these patients with neuroblastoma^11^ and in up to two-thirds of those with a family history of WT. Furthermore, an underlying genetic susceptibility can be found in 10% of apparently sporadic WT.^8^

*MYCN* encodes the MYCN protein, a proto-oncogenic MYC family transcription factor. *MYCN* is frequently somatically altered in both neuroblastoma and WT (and more rarely in a range of other childhood tumours). In neuroblastoma, it is amplified in up to 25% of cases. *MYCN* amplification is a criterion defining high-risk disease (except in the unusual circumstances of a localised, resectable tumour with *MYCN* amplification, when it is treated as intermediate-risk).^12^ WT has a more heterogeneous range of *MYCN* alterations that can indicate poorer prognosis, including gain, hotspot point mutations and hypomethylation of specific loci leading to over-expression of MYCN.^13^

Germline 2p24.3 duplication has been observed in several cases of Wilms Tumour and neuroblastoma, either as part of a larger 2p duplication,^14^, ^15^, ^16^, ^17^ as a microduplication just involving 2p24.3, in 5 cases in 4 families,^17^, ^18^, ^19^, ^20^ or an intermediate length 8.6 Mb duplication including 2p24.3 in one child with neuroblastoma.^21^ The study by Gillani et al. found 2p24 *de novo* germline/mosaic post-zygotic duplications, including *MYCN* in 2 out of 690 children with neuroblastoma, and none in the 8683 other participants who were either children with osteosarcoma or Ewing Sarcoma, unaffected parents or adult population controls. Although the larger duplications involve many genes, including *ALK*, the microduplications have been localised to a region including *MYCN* and *DDX1*. It is present in gnomAD 4.1.0 with an allele frequency of 1/125,352.^22^ As all reported microduplications involved both the *MYCN* and *DDX1* genes, it has not been possible to know which was responsible for any predisposition. Interestingly, all the cases of neuroblastoma with germline microduplications, including *MYCN,* have been found to have somatic *MYCN* high-level amplification.

DDX1 is a DEAD box family protein involved in DNA repair and RNA processing, and its gene is located 306 kb from *MYCN* in a telomeric direction. It is co-amplified in 50–70% of *MYCN*-amplified neuroblastomas and may lead to increased dependency on the mTOR pathway,^23^ although the effect on prognosis, if any, is controversial.^24^^,^^25^

Due to the frequent somatic changes in *MYCN* and the clear association with prognosis when amplified, it has been felt most likely that any increased risk of cancer in those with a 2p24.3 duplication was due to the gain of *MYCN* rather than the presence of *DDX1* within the region of duplication, but this has not been proven. As described in the recent AACR Childhood Cancer predisposition paper on neuroblastoma, the population prevalence of 2p24.3 microduplications and the penetrance are currently unknown.^26^

We sought to systematically understand the prevalence of 2p24.3 germline microduplications that include *MYCN* and/or *DDX1*, and the incidence of childhood malignancy in carriers by interrogating a large, national Whole Genome Sequencing library.

## Methods

### Participant cohort

Participants consented to participation in the National Genomic Research Library (NGRL),^27^ either from enrolment in the 100,000 Genomes Project (100kGP) or following sequencing as part of the United Kingdom National Health Service Genomic Medicine Service (NHS GMS). The 100kGP ran between 2015 and 2018, recruited participants with either a diagnosis of cancer or an undiagnosed rare disease, and all underwent Whole Genome Sequencing (WGS). Since 2021, eligible patients have had WGS via the NHS GMS clinical pathway. Eligibility criteria included many rare diseases and several cancer indications; in particular, all patients with cancer aged under 25 years at diagnosis were eligible. Participants with cancer had both somatic and germline samples sequenced. Probands with rare diseases had germlines sequenced, as did their close family members when feasible. Linked clinical data from Hospital Episode Statistics and the National Cancer Registry and Analysis Service were available for both cancer and rare disease participants in the 100kGP. Human Phenotype Ontology terms were available for those with rare diseases in both 100kGP and GMS. Any phenotypes present in <5 participants within the NGRL are reported as <5, in accordance with the Genomics England data security policy.^28^ Neuroblastoma is used as an umbrella term to describe both neuroblastoma and peripheral neuroblastic tumours, such as ganglioneuroblastoma and ganglioneuroma. Data governance requirements do not allow us to identify if any of the participants in this study have been included in previously published reports.

### Sequencing pipeline

Sample processing is as previously published.^29^^,^^30^ 100kGP cancer samples had undergone Illumina's North Star sequencing pipeline (version 2.6.53.23). Read alignment against the human reference genome GRCh38 (with decoy and EBV contigs) had been performed with ISAAC (version 03.16.02.19). Structural variants (SVs) and long indel (>50 bp) had been called using Manta (version 0.28.0), and Copy Number Variants (CNVs) had been called with Canvas (version 1.3.1).

GMS samples had been passed through the DRAGEN pipeline. Samples had been aligned using DRAGEN (version 3.2.22). 100kGP cancer genomes had also been reanalysed using this pipeline. DRAGEN had also been used for germline SV calling, Canvas (1.39) for somatic CNV and Manta (version 1.5) for somatic SVs.

### Structural variant workflow

The Genomics England Structural Variant workflow v3.0 has been developed to extract SV and CNV from structural vcf files for specified participants, and it provides an output of any SV or CNV that overlap or include a specified region. It is written in Nextflow DSL2 (version 22.10.5) and utilises containerised R (version 4.2.1) (and the RLabKey API), Python 3 (version 3.11.4) and bcftools (version 1.16). For this project, it was supplied with region coordinates spanning a region from 70 kb in a telomeric direction of the *MYCN* locus (chromosome 2: 15940550–15947007 using the latest GRCh38 assembly) to 70 kb in a centromeric direction from the *DDX1* locus (chromosome 2: 15591178–15634346). It was run on the entire 100kGP rare disease germline cohort, 100kGP cancer cohort and entire GMS cohort. SV results were filtered to include duplications of between 2 kb and 20 Mb, including the *MYCN* or *DDX1* loci, as were CNV gains that included the *MYCN* or *DDX1* loci, and then manually inspected in Integrative Genomics Viewer (IGV) version 2.15.4. Split alignment reads were analysed using BLAT,^31^ which confirmed the tandem duplication breakpoints. UCSC Genome Browser was used for visualisation.^32^

### Statistical analysis

Statistical analysis was performed in R (v 4.2.1). Associations were assessed using Fisher's exact test (two-sided), with a p-value of <0.05 deemed significant. An estimate of penetrance was carried out by Bayesian calculation of the conditional probability of disease using the following formula,^33^ where D is the presence of Wilms Tumour or neuroblastoma, G is the presence of microduplication that includes *MYCN* and $\bar{D}$ is the absence of Wilms Tumour or neuroblastoma:

$$P\left(D|G\right)=\frac{P\left(G|D\right)\;\times \;P\left(D\right)}{P\left(G|D\right)\;\times \;P\left(D\right)\;+\;P\left(G|\bar{D}\right)\;\times \;P\left(\bar{D}\right)}$$

Confidence interval for the penetrance estimate was calculated using the Clopper-Pearson exact method to estimate 95% confidence intervals for the frequency of the microduplication, including *MYCN*, in cases (P(GǀD)) and controls (P(Gǀ$\bar{D}$)), and then using these upper and lower bounds of the confidence intervals to calculate upper and lower estimates of the penetrance. For the upper bound of the penetrance confidence interval the upper bound for P(GǀD) and the lower bound for P(Gǀ$\bar{D}$) are used, and for the lower bound of the penetrance confidence interval the lower bound for P(GǀD) and the upper bound for P(Gǀ$\bar{D}$) are used. By using the respective pairs of bounds for both P(GǀD) and P(Gǀ$\bar{D}$), we ensure that the coverage of the confidence interval for the estimation of penetrance will be in excess of 95%.

### Ethics

The National Genomics Research Library has been established following East of England–Cambridge Central Research Ethics Committee approval 20/EE/0035. This research was conducted in line with Genomics England data security and governance policies. All participants (or their parents/guardians) had provided informed consent to participate in the NGRL. In line with the ethics approval, we did not separately seek consent for this study, as the data included was approved for use by the Genomics England airlock process.

### Role of funders

The funders of this study had no role in study design, data collection, data analysis, interpretation, or writing of the report.

## Results

### Patient cohort

**Fig. 1:**
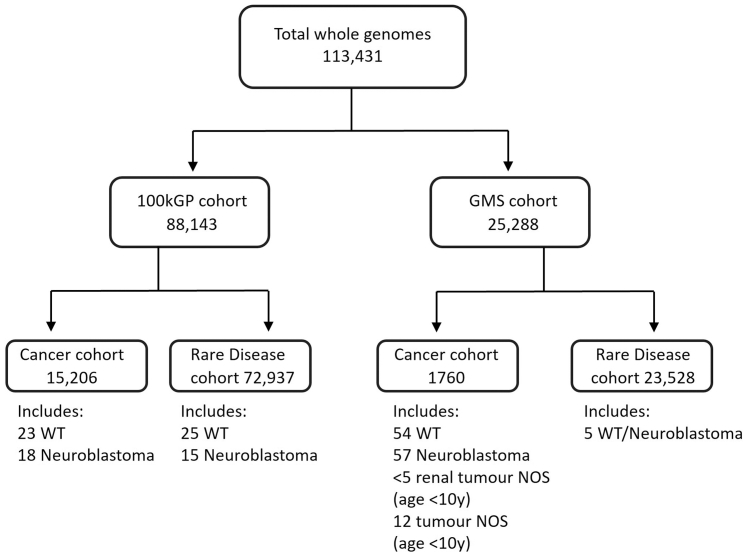
Participants included in study cohort by recruitment cohort and indication for whole genome sequencing. Abbreviations: 100kGP, 100,000 Genomes Project; GMS, Genomic Medicine Service; WT, Wilms Tumour; NOS, not otherwise specified.

The National Genomic Research Library had 113,431 genomes available for analysis in the NGRL (Fig. 1). This included 88,143 participants recruited to the 100kGP (main programme data release version 17), including 15,206 whose indication for WGS was cancer, who had both somatic and germline sequences available, and 72,937 who had WGS because of a rare disease, who had germline sequencing only. Of those recruited for cancer, 23 had a WT and 18 had neuroblastoma. Of those recruited for rare disease, 25 had a history of WT or developed one subsequently, and 15 had a history of neuroblastoma or developed one subsequently, and this was amongst other diagnoses/physical signs that indicated a possible underlying syndrome for all but six. Nine of the patients in the 100kGP rare disease arm with WT or neuroblastoma received a genetic diagnosis that explained some or all of their features. An additional 25,305 genomes had been sequenced as part of the Genomic Medicine Service (GMS data release version 3), including 23,545 who had germline sequencing for rare disease, of whom five had a history of either WT or neuroblastoma, all of whom had additional features that had suggested a possible syndrome. 1760 were sequenced because of a diagnosis of cancer, with both somatic and germline sequences available. This included 54 with WT, 57 with neuroblastoma, <5 with an unspecified renal tumour in children aged <10 years, and 12 children aged <10 years with an unspecified tumour type.

### 2p24.3 microduplication that includes *MYCN*

The structural variant workflow identified 6 unique participants across the combined cohort with a microduplication that included the *MYCN* locus (Table 1). Of these participants, two had a diagnosis of WT (one bilateral, one unilateral), and one had a diagnosis of neuroblastoma. One participant recruited to the cancer arm was additionally sequenced as a trio as part of the rare disease arm and was, therefore, identified twice by the workflow. They are counted only once in the following analyses. None of the patients with WT or neuroblastoma had documented dysmorphic features or neurodevelopmental delay.

**Table 1:** Participants identified with a 2p24.3 microduplication that included *MYCN* locus.

Participant	5′ Breakpoint	3′ Breakpoint	Genes included	Childhood tumour	Somatic MYCN change	Other somatic drivers
MYCN_01	15,514,487	16,012,319	Part of *NBAS, DDX1, LINC01804, MYCN, MYCNOS*	Bilateral WT	*MYCN* gain	Nil
MYCN_02	15,478,041	16,159,837	Part of *NBAS, DDX1, LINC01804, MYCN, MYCNOS*, part of *GACAT3*	Unilateral WT	*MYCN* gain	CNLOH 11p Loss of 16q Gain of 1q Deletion of exon 1 *AMER1*
MYCN_03	15,846,220	17,332,343	*MYCN, MYCNOS, GACAT3, FAM49A*	Neuroblastoma	*MYCN* amplification	Hotspot *ALK* SNV Gain of 6q, 17q Loss of 1p, 10q
MYCN_04	15,514,487	16,012,319	Part of *NBAS, DDX1, LINC01804, MYCN, MYCNOS*	No tumour	N/A	N/A
MYCN_05	15,629,926	16,071,956	Part *of DDX1, LINC01804, MYCN, MYCNOS,* part of *GACAT3*	No tumour	N/A	N/A
MYCN_06	15,366,588	16,023,870	Part of *NBAS, DDX1, LINC01804, MYCN, MYCNOS*	No tumour	N/A	N/A

Abbreviations: CNLOH, Copy Neutral Loss of Heterozygosity; SNV, Single Nucleotide Variant.

Of the three participants who did not have a history of a childhood embryonal tumour, one had only a subtle copy number rise and was likely mosaic for the duplication, and was sequenced as an unaffected parent of one of the participants with a childhood tumour. Of the other two, one had an enrolment diagnosis of breast cancer, and the other had developmental delays and multiple dysmorphisms. Clinical data were available until the age of nine years for one patient and adulthood for two.

A 2p24.3 microduplication that included *MYCN* was therefore identified in 3/197 with a definite history of WT/neuroblastoma and 3/113,234 without a known history of WT/neuroblastoma (Fisher's exact test statistic value is < 0.0001).

### Estimate of the penetrance of 2p24.3 microduplication that includes *MYCN*

Using published rates of childhood Wilms Tumour^4^ and neuroblastoma^34^ in High-Income Countries, we estimated a combined incidence of these two tumours during childhood as 2.7/10,000. We used the results from this study to estimate the population prevalence of the 2p24.3 microduplication that includes *MYCN* as being 3/113,234. These were then used to estimate penetrance of 13% (95% CI, 2%–60%) for developing WT/neuroblastoma if there is a germline 2p24.3 microduplication that includes *MYCN*.

### Somatic *MYCN* changes

**Fig. 2:**
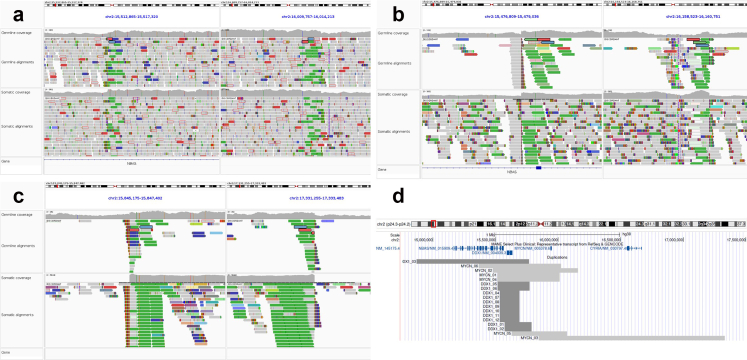
a–c: IGV images showing germline and somatic coverage and alignments for each tumour associated with germline 2p24.3 microduplication that included *MYCN*. (a and b) are sequences from participants who developed a WT. (c) sequences from a participant who developed neuroblastoma. (d): UCSC Genome Browser image showing all participants with a 2p24.3 duplication including *MYCN* and/or *DDX1*.

All three participants with 2p germline gain and a childhood embryonal tumour had a somatic sequence available for analysis. The two participants with Wilms Tumour each had *MYCN* gain in their tumours, as in the germline (Fig. 2a and b). The participant with neuroblastoma had an amplification (>50 copies) of *MYCN*, with breakpoints corresponding to the region of gain in the germline (Fig. 2c).

### Additional somatic changes

WGS of tumour samples in the participants with either a WT or neuroblastoma identified no additional somatic driver for one participant with WT. The other WT had common WT somatic drivers including copy-neutral loss of heterozygosity of 11p, heterozygous loss of 16q, gain of 1q and a disrupting deletion in *AMER1* leading to deletion of exon 1. The neuroblastoma had an activating hotspot *ALK* single nucleotide variant (p.Phe1174Leu) and segmental chromosomal abnormalities including heterozygous loss of 1p and 17q, and gain of 6q, 17q (Table 1).

### Microduplication that includes *DDX1* but not *MYCN*

Previous case reports of 2p24.3 microduplications and embryonal tumours have all included both the *MYCN* and *DDX1* loci within the area of microduplication, and it was, therefore, not possible to distinguish if the *DDX1* gene, *MYCN* gene or both were responsible for the predisposition to embryonal tumours. We therefore sought to identify participants with microduplications that included the *DDX1* locus but not *MYCN*.

Twelve participants were identified with a microduplication that included the *DDX1* locus but not the *MYCN* locus (Table 2). None of these participants had received a diagnosis of a childhood embryonal tumour. Six had been recruited for indication of rare disease (one with ataxia, one with a connective tissue disorder, one with visual impairment, one with a history of familial breast cancer, one with muscular dystrophy and one with developmental delay), five were unaffected parents, and one had colon cancer. Among the 12 participants, there were four pairs where an unaffected parent and a proband both had the duplication, three probands who did not have trio testing and an unaffected parent whose affected relative did not have the duplication. In no cases had the *DDX1* duplication been interpreted by their clinical team as causative for a phenotype. For four of the six participants with a rare disease, an alternative genetic diagnosis for their phenotype had been identified through WGS. Seven participants had a duplication with the same breakpoints, and a region of GGAG microhomology was identified at each breakpoint. Of these patients, clinical follow-up data was available until the age of seven years for a single patient, age 11 years for two patients, adulthood for eight patients and one patient died aged <5 years. Only the microduplication for participant DDX1_03 was present in gnomAD 4.1.0 with an allele frequency of 1/126,092.^22^

**Table 2:** Participants identified with a 2p24 microduplication that included *DDX1* locus but not *MYCN* locus.

Participant	5′ Breakpoint	3′ Breakpoint	Genes included	Childhood tumour
DDX1_01	15,572,502	15,785,894	*DDX1, LINC01804*	No tumour
DDX1_02	15,572,502	15,785,894	*DDX1, LINC01804*	No tumour
DDX1_03	14,869,110	15,767,826	*NBAS, DDX1, LINC01804*	No tumour
DDX1_04	15,535,429	15,691,141	Part of *NBAS*, *DDX1,* Part of *LINC01804*	No tumour
DDX1_05	15,515,999	15,770,440	Part of *NBAS*, *DDX1*, *LINC01804*	No tumour
DDX1_06	15,515,999	15,770,440	Part of *NBAS*, *DDX1*, *LINC01804*	No tumour
DDX1_07	15,535,429	15,691,141	Part of *NBAS*, *DDX1,* Part of *LINC01804*	No tumour
DDX1_08	15,535,429	15,691,141	Part of *NBAS*, *DDX1,* Part of *LINC01804*	No tumour
DDX1_09	15,535,429	15,691,141	Part of *NBAS*, *DDX1,* Part of *LINC01804*	No tumour
DDX1_10	15,535,429	15,691,141	Part of *NBAS*, *DDX1,* Part of *LINC01804*	No tumour
DDX1_11	15,535,429	15,691,141	Part of *NBAS*, *DDX1,* Part of *LINC01804*	No tumour
DDX1_12	15,535,429	15,691,141	Part of *NBAS*, *DDX1,* Part of *LINC01804*	No tumour

## Discussion

We have demonstrated that patients with microduplications of the *MYCN* locus have a raised incidence of childhood WT and neuroblastoma and that these microduplications are extremely rare in the general population. We estimated the penetrance to be 13% based on our data. This would be in line with many other Wilms Tumour and neuroblastoma predisposition genes, which lead to increased risk for these embryonal tumours during a narrow window of development in early childhood. Although there are some predisposition genes with a very high penetrance (such as most *WT1* variants for WT predisposition or ALK activating variants for neuroblastoma both with penetrance of ≥50%), there are many with intermediate penetrance where screening is recommended, such as Simpson-Golabi Behmel syndrome from *GPC3/4* variants (estimated 5% risk of WT and 5% of hepatoblastoma), Beckwith-Weidemann Syndrome (estimated WT risks of <1–20% depending on genetic aetiology), Bohring-Opitz Syndrome from ASXL1 variants (estimated WT risk of 7%) and PHOX2B variants (estimated risk of neuroblastoma of 5%).

We have shown that although *DDX1* had previously been implicated as potentially involved, there does not appear to be any increased incidence of childhood tumours when *DDX1* alone is duplicated. This supports the hypothesis that it is *MYCN* that is responsible for the excess risk in patients with 2p gains. We would not recommend that the presence of a *DDX1* duplication without *MYCN* duplication be an indication for surveillance or screening.

All the unrelated participants identified in this study with a duplication, including *MYCN*, had different breakpoints. There were no consistent regions of microhomology that suggested the mechanism for this duplication. The germline duplication appears to be an intrachromosomal tandem duplication. Intriguingly, the somatic amplification shares the exact same breakpoints as the germline duplication. Early FISH studies of *MYCN* amplification in neuroblastoma cell lines reported deletion of the *MYCN* sequence from one chromosome 2, which corresponded precisely to the amplified sequence on the other chromosome 2, which is now a recognised feature of extrachromosomal amplifications.^35^ The shared breakpoint suggests the duplication predisposes to a structural mechanism of amplification (for example, non-allelic homologous recombination with additional risk via the additional copy of the tandem breakpoint) and not just a biological one caused by over-expression of *MYCN*. Further work is planned to confirm if the case with subsequent somatic amplification of this region has an intrachromosomal amplification or is present as extrachromosomal DNA. Both mechanisms have been reported in sporadic neuroblastoma with *MYCN* amplification, although extrachromosomal DNA is more common in tumours, and intrachromosomal amplification is more common in cell lines. Understanding the mechanism of amplification in these cases with germline duplication might provide insights into the mechanisms in sporadic cases.

A strength of this study is that we were able to apply a consistent pipeline across a very large cohort of participants from many hospitals across the United Kingdom, but who were sequenced centrally with harmonised sample processing and bioinformatics pipelines. This gives confidence to our assertion that this duplication is very rare in the general population. Limitations of this include the relatively small number of patients with a history of WT or neuroblastoma, and, having shown the rarity of this duplication in the general population, follow-up studies in larger disease-specific cohorts may be warranted, and the potential for bias, as most participants were recruited either for cancer or rare disease.

For patients with genetic predispositions to WT and/or neuroblastoma, surveillance is recommended during the crucial period of development, where neuroblastoma and WT are most likely to develop. When WT screening is recommended, both the SIOP Renal Tumour Working group^36^ and AACR^9^ suggest this is done with three monthly renal ultrasound scans from birth to the age of seven years. Neuroblastoma predisposition screening recommendations vary, but the current AACR recommendation^26^ is for screening with abdominal US, urine HVA/VMA and chest radiographs. This is done three monthly from birth to the age of six years, and six monthly from six to the age of ten years. The intention would be that earlier diagnosis would enable detection of lower stages at diagnosis and hopefully improve survival. For patients with WT, the lower stage is also of vital importance for increasing the chances of successful nephron-sparing surgery in cases where bilateral WT develops. We recommend 2p24.3 microduplications that include *MYCN* should be considered for inclusion in germline childhood cancer predisposition panels for routine assessment when a WT or neuroblastoma predisposition is suspected.

## Contributors

Catherine A. Taylor -conceptualisation, literature search, data analysis, data interpretation, writing.

Philippa May—data analysis, data interpretation, writing—review and editing.

Thomas J. Stone—data analysis, writing—review and editing.

Munaza Ahmed—data interpretation.

Tanzina Chowdhury–data interpretation, writing—review and editing.

Deborah Tweddle–data interpretation, writing—review and editing.

Shaun Wilson–data interpretation.

Ken Hanscombe–software.

J. Ciaran Hutchinson—data analysis, data interpretation.

Jessica C. Pickles—supervision, data analysis, data interpretation, writing—review and editing.

Neil J. Sebire–supervision.

Thomas S. Jacques—supervision, study design, data interpretation, writing—review and editing.

All authors read and approved the final version of the manuscript. All underlying data was accessed and verified by authors, including Catherine Taylor and Philippa May.

## Data sharing statement

Research on the de-identified patient data used in this publication can be carried out in the Genomics England Research Environment subject to a collaborative agreement that adheres to patient-led governance. All interested readers will be able to access the data in the same manner that the authors accessed the data. For more information about accessing the data, interested readers may contact research-network@genomicsengland.co.uk or access the relevant information on the Genomics England website: https://www.genomicsengland.co.uk/research.

## Declaration of interests

CT was supported by a PhD studentship from the UCL Great Ormond Street Institute of Child Health Child Health Research CIO. NS and TJ were supervisors of this studentship. JCP is funded by a “New Roads” grant from Cancer Research UK. JCH has received payment for expert testimony from the UK Crown Prosecution Service and support to attend meetings from Cirdan Imaging Ltd. SW has received consulting fees from Norgine and honoraria for lecture from Bayer. TJ has grants from the Brain Tumour Charity, Cancer Research UK and the Olivia Hodson Cancer Fund; has received consulting fees from the National Institute of Clinical Excellence; and is on the expert editorial panel of the WHO Classification of Tumours and a member of the cIMPACT-NOW consortium; and was the lead for the Children's Solid Tumour domain of the Genomics England Clinical Interpretation Partnership (GeCIP). KH is employed by Genomics England Ltd. DT has received conference travel support from Recordati Rare Diseases. The other authors have no relevant declarations of interest.
