# Supplementary material for: Germline duplication of MYCN predisposes to childhood embryonal tumours
Source: eBioMedicine. 2026 Jan 31;124:106132. doi: 10.1016/j.ebiom.2026.106132 (PMC12878678; doi:10.1016/j.ebiom.2026.106132)
Supplement: Supplementary Material [file mmc1.docx]

Supplementary Results for Germline duplication of *MYCN* predisposes to childhood embryonal tumours

Catherine A. Taylor, Philippa May, Thomas J. Stone, Munaza Ahmed, Tanzina Chowdhury, Deborah A. Tweddle, Shaun Wilson, Ken Hanscombe, J. Ciaran Hutchinson, Jessica C. Pickles, Neil Sebire, Thomas S. Jacques

Calculations of penetrance

The formula used for the Bayesian calculation of the conditional probability of disease:

$$Penetrance=\frac{P\left( G | D \right) \times P(D)}{P\left( G | D \right)\times P\left( D \right)+P\left( G | \bar{D} \right) \times P(\bar{D})}$$

For the sample in this study:

$P\left( G | D \right)$ = 3/197 = 0.01523

$P\left( D \right)$ = 2.7/10000 = 0.00027

$P\left( G | \bar{D} \right)$ = 3/113234 = 0.000026

$P\left( \bar{D} \right)$ = 9997.3/10000 = 0.9997

$$Penetrance=\frac{P\left( G | D \right) \times P\left( D \right)}{P\left( G | D \right)\times P\left( D \right)+P\left( G | \bar{D} \right) \times P\left( \bar{D} \right)}=0.1344$$

In order to calculate the 95% Confidence Interval for the penetrance, first the 95% Confidence intervals for case and control genotype frequencies were calculated using Clopper-Pearson exact method:

95% Confidence intervals for case genotype frequency $(P\left( G | D \right))$:

Lower 95% CL: 0.003152

Upper 95% CL: 0.04386

95% Confidence intervals for control genotype frequency $(P\left( G | \bar{D} \right))$:

Lower 95% CL: 0.000007742

Upper 95% CL: 0.00005475

For calculating upper bound of the confidence interval, using upper bound of $P\left( G | D \right)$ = 0.04386 and the lower bound for $P\left( G | \bar{D} \right)$ = 0.000007742,

$$\frac{P\left( G | D \right) \times P\left( D \right)}{P\left( G | D \right)\times P\left( D \right)+P\left( G | \bar{D} \right) \times P\left( \bar{D} \right)}=0.6047$$

For calculating lower bound of the confidence interval, using lower bound of $P\left( G | D \right)$ = 0.003152 and the upper bound for $P\left( G | \bar{D} \right)$ = 0.00005475,

$$\frac{P\left( G | D \right) \times P(D)}{P\left( G | D \right)\times P\left( D \right)+P\left( G | \bar{D} \right) \times P(\bar{D})}=0.01531$$
